# Lessons learned from a SIRT2-selective inhibitor

**DOI:** 10.18632/oncotarget.8502

**Published:** 2016-03-30

**Authors:** Hui Jing, Hening Lin

**Affiliations:** Howard Hughes Medical Institute, Department of Chemistry and Chemical Biology, Cornell University, Ithaca, NY, USA

**Keywords:** sirtuin, Myc, oncoprotein, COMPARE analysis, targeted cancer therapy

**Figure 1:**
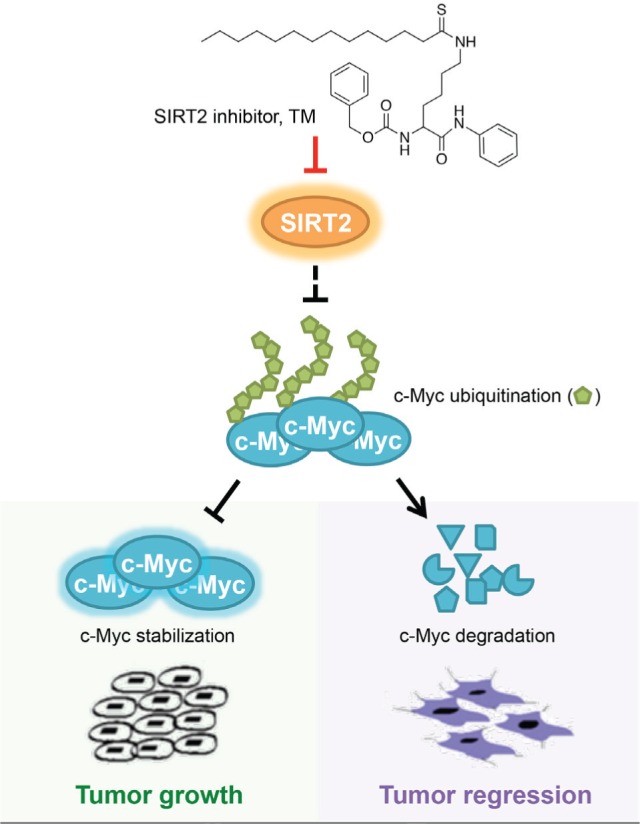
SIRT2-selective inhibitor TM represses tumor cell growth by promoting the proteolytic degradation of Myc.

Developing targeted cancer therapies typically starts with the identification of oncoproteins that are mutated or over-activated in tumors, followed by the development of small molecules that target the oncoproteins. A paradigm example is the development of Gleevec targeting the BCR-ABL oncoprotein in chronic myeloid leukemia. Many other targeted therapies follow this logic, such as compounds that target RAS mutants or overexpressed Myc (reviewed in [1]). We recently developed a potent and selective SIRT2 inhibitor, TM, which exhibits broad anticancer activity partly by decreasing the protein level of Myc (Figure 1) [2]. Here we summarize several lessons we learned from this study with the intention that they may help other researchers developing targeted cancer therapies.

**The Achilles heel of cancer is not necessarily the driver of cancer (oncoproteins can be targeted indirectly).** When developing anticancer therapeutics, it is natural to think of targeting oncoproteins that cause tumors. Myc is one oncoprotein that is highly pursued as a cancer target. Despite intensive studies, directly targeting Myc remains challenging. However, several bromodomain inhibitors show promising anticancer activity and decrease Myc transcription [3]. MEK/ERK and PI3K/AKT signaling inhibitors may also modulate Myc protein stability for therapeutic benefits [3]. Our studies suggest that SIRT2 inhibition is another way to indirectly target Myc. TM upregulates the transcription of several Myc E3 ubiquitin ligases that promote Myc degradation, and thus inhibits cancer cells. Interestingly, SIRT2 level in cancer cell lines does not correlate with the sensitivity of these cell lines to TM. Furthermore, TM does not decrease Myc level in normal cells. Therefore, the Achilles heel of cancer (SIRT2 in this case) does not have to be the driver (upregulated Myc) of cancer. Cancer cells may have unique vulnerabilities, other than the oncoproteins, that can be targeted while sparing normal cells.

**Phenotype of genetic knockout mice may not predict the effect of small molecule inhibitors.** SIRT2 belongs to the sirtuin family of protein lysine deacylases, which play important roles in many biological pathways. There is sustained interest in exploring whether sirtuins can be therapeutic targets for human cancers. However, a previous study showed that *Sirt2* knokcout mice tend to develop tumors earlier than wildtype mice [4]. So we were surprised to find that the SIRT2-selective inhibitor, TM, inhibits the proliferation of many cancer cell lines. We confirmed with several experiments that SIRT2 inhibition does produce the anticancer effects. This experience suggests that the phenotype of genetic knockout mice may not predict the effect of small molecule inhibitors. Getting potent and selective inhibitors is the ultimate way to demonstrate whether or not a protein is an ideal anticancer target.

**Fundamental understanding of the underlying biomolecules is important for developing new therapeutic leads.** TM, with the best combination of potency and selectivity among known SIRT2 inhibitors, was developed through fundamental understandings of the enzymatic activity of sirtuins. Sirtuins were originally thought to only remove the two-carbon acetyl groups from protein lysine residues. Recent studies reveal that several sirtuins with weak deacetylase activities can efficiently remove other acyl groups. For example, SIRT5 is an efficient desuccinylase and SIRT6 efficiently removes long chain fatty acyl groups. More surprisingly, even the sirtuins with strong deacetylase activity, SIRT1-3, can remove long chain fatty acyl groups efficiently *in vitro* (reviewed in [5]). These findings enabled us to develop TM as a potent and selective SIRT2 inhibitor. Therefore, the fundamental understanding about the sirtuin enzymatic activities drove the development of the SIRT2 inhibitor, which in turn led to the translational research on the development of anticancer therapeutics. The importance of basic research is thus emphasized once again.

**NCI-60 screening and COMPARE analysis are useful tools to investigate the mechanism of action of anticancer agents.** After discovering TM's anticancer activity, we wished to understand how SIRT2 inhibition cripples cancer cells. SIRT2 has many known functions that could explain the anticancer effect of TM. For example, SIRT2 is reported to deacetylate tubulin (a protein important for mitosis), deacetylate APC/C (a complex that is important for cell cycle), destabilize Myc, and activate LDH-A (lactate dehydrogenase A, which contributes to the Warburg effect) (reviewed in [6]). It was not clear disrupting which of these functions is important for the anticancer effect. We submitted TM to the Development Therapeutic Program at the National Cancer Institute (NCI), where TM was screened in the NCI-60 panel of human cancer cell lines. NCI has a lot of data on the NCI-60 cell lines, such as sensitivity to drug molecules, mRNA/protein levels, and posttranslational modifications. We used the COMPARE analysis [7] to correlate the sensitivity of the NCI-60 cells to TM with the molecular properties of these cell lines. We found that the sensitivity of NCI-60 cell lines to TM correlated best with Myc phosphorylation/protein levels, which led us to identify Myc degradation as an important mechanism by which TM inhibits cancer cells. Thus, NCI-60 screening combined with COMPARE analysis is an effective way for discovering the mechanism of anticancer agents.

Our finding that SIRT2-specific inhibitors are promising therapeutic candidates against Myc-driven cancers also raised many questions. For instance, it is unclear how SIRT2 regulates the expression of E3 ligases of Myc. Also, SIRT2 possesses both deacetylase and defatty-acylase activities. It will be interesting to differentiate the contribution of SIRT2's defatty-acylase activity in cancers from that of the deacetylase activity. Despite these questions, the above lessons we have learned from this project can be useful to other researchers interested in developing anticancer therapeutics.
